# UK Healthcare Professionals’ Knowledge, Attitudes, and Beliefs About Adult Obesity: A Qualitative Study

**DOI:** 10.1002/osp4.70182

**Published:** 2026-08-04

**Authors:** Paul MacDonald, Helen Frank, Rebecca Stack

**Affiliations:** ^1^ School of Health and Wellbeing University of Worcester Worcester UK

**Keywords:** attitudes, beliefs, COSMIN, healthcare professionals, knowledge, obesity, outcome measurement instrument, qualitative research

## Abstract

**Background:**

Healthcare professionals’ knowledge, attitudes, and beliefs toward adults living with obesity influence clinical engagement, communication, and quality of care. However, existing outcome measurement instruments used to assess these constructs have demonstrated limitations in development quality, contextual relevance, and content validity, particularly within the United Kingdom (UK) healthcare system.

**Objective:**

To conduct a qualitative concept elicitation study aligned with the Consensus‐based Standards for the Selection of Health Measurement Instruments (COSMIN) recommendations to identify key themes shaping UK healthcare professionals’ knowledge, attitudes, and beliefs regarding adults living with obesity in order to inform the development of an outcome measurement instrument.

**Design:**

Qualitative descriptive study using semi‐structured group and individual interviews.

**Methods:**

35 UK healthcare professionals from multiple disciplines participated in semi‐structured individual and group interviews. Data were analysed using inductive reflexive thematic analysis in accordance with COSMIN recommendations for content validity and item generation.

**Results:**

Four interrelated themes were identified: (1) clinical working environment, (2) knowledge of obesity, (3) attitudes to obesity, and (4) beliefs about professional roles. The clinical working environment was identified as a primary contextual determinant influencing how knowledge is constructed, how attitudes are expressed, and how beliefs about professional roles are enacted in practice. Structural constraints, organizational culture, role ambiguity, and service limitations influenced clinicians' confidence, communication, and perceived legitimacy in adult obesity care.

**Conclusion:**

Healthcare professionals' knowledge, attitudes, and beliefs regarding adult obesity are not solely individual attributes but are embedded within organizational and service contexts. These findings provide a strong empirical foundation for COSMIN‐aligned item development and support the creation of a context‐sensitive outcome measurement instrument to inform research, workforce development, and service improvement in UK adult obesity care.

**Patient or Public Contribution:**

Patient and Public Involvement (PPI) contributors reviewed the interview guide to ensure clarity and relevance prior to data collection.

## 1 Introduction

Adult obesity poses a substantial public health challenge in the United Kingdom (UK), affecting more than one in four adults and contributing to significant long‐term morbidity, increased healthcare utilization, and economic burden [1, 2]. Effective obesity management depends not only on clinical guidelines and service provision but also on healthcare professionals' knowledge, attitudes, and beliefs, which influence communication, clinical decision‐making, and patient engagement [3, 4, 5, 6, 7, 8, 9, 10, 11].

Although national guidance, including the National Institute for Health and Care Excellence (NICE) guideline CG189, provides evidence‐based recommendations for obesity management in the UK [7], previous research consistently demonstrates variations in healthcare professionals’ knowledge, attitudes, and beliefs, including uncertainty, weight stigma, and inconsistent confidence in obesity‐related care [8, 9, 10, 11]. These factors may adversely affect clinical interactions, patient engagement, and the delivery of evidence‐based obesity care.

Understanding healthcare professionals’ knowledge, attitudes, and beliefs requires robust outcome measurement instruments. However, the validity of these instruments depends upon clear conceptual definitions and strong content validity. A recent Consensus‐based Standards for the Selection of Health Measurement Instruments (COSMIN) based scoping review evaluated existing outcome measurement instruments designed to assess healthcare professionals’ knowledge, attitudes, and beliefs regarding adult obesity and identified important methodological limitations, including poor content validity, conceptual ambiguity, limited relevance to the UK context, and inadequate differentiation between the underlying constructs [12, 13, 14, 15, 16, 17, 18]. Consequently, confidence in the existing outcome measurement instruments remains limited. While the scoping review evaluated the methodological quality of existing outcome measurement instruments, the present study identifies the concepts that should be measured through qualitative concept elicitation, thereby providing the empirical foundation for COSMIN‐compliant item development.

Although the scoping review identified significant weaknesses in existing outcome measurement instruments, it did not identify which concepts should be measured. In accordance with COSMIN guidance, establishing content validity necessitates qualitative concept elicitation from the target population prior to item generation or psychometric testing [12, 13, 19]. Therefore, before a valid outcome measurement instrument can be developed, it is essential to understand how healthcare professionals conceptualize obesity, how these concepts are articulated in clinical practice, and which contextual factors influence them.

This study represents the concept elicitation phase of a broader research programme aimed at developing an outcome measurement instrument for healthcare professionals’ knowledge, attitudes, and beliefs regarding adult obesity. The instrument is intended for initial use within UK healthcare settings, with potential adaptation and validation for broader international applications. Through qualitative interviews with healthcare professionals from multiple disciplines, the study identified the core conceptual themes and contextual influences underpinning these constructs, thereby providing an empirical foundation for future item generation and psychometric evaluation.

## 2 Methods

### 2.1 Study Design

This study employed a qualitative descriptive design, underpinned by a pragmatic research paradigm, to undertake concept elicitation in accordance with the Consensus‐based Standards for the Selection of Health Measurement Instruments (COSMIN) recommendations for content validity. Pragmatism was selected because it supports knowledge generation that is directly applicable to the development of early‐phase outcome measurement instruments. This study aimed to identify the conceptual themes underpinning healthcare professionals' knowledge, attitudes, and beliefs regarding adults living with obesity to inform the development of a content‐valid outcome measurement instrument.

Semi‐structured group and individual interviews were conducted between May and June 2025. Both individual and group interviews were used to capture personal experiences alongside shared professional perspectives across different healthcare settings. Thirty‐five healthcare professionals participated, comprising three group interviews (*n* = 6, *n* = 6, *n* = 4) and 19 individual interviews. All interviews were conducted virtually using Microsoft Teams, lasted up to 60 minutes, and followed a standardized interview guide. Ethical approval was obtained from the University of Worcester Health and Research Ethics Committee (Reference: HS25260014).

### 2.2 Participants and Recruitment

Purposive sampling was used to recruit healthcare professionals across a range of professional roles, clinical disciplines, and healthcare settings within the UK to reflect the intended cross‐professional application of the planned outcome measurement instrument. Participants were eligible for interview if they were registered with a recognized UK regulatory body (e.g., HCPC, GMC, NMC), were actively engaged in clinical practice, were involved in direct patient care, and were proficient in English.

Recruitment was undertaken through professional networks, NHS Research and Development departments, which acted as organizational gatekeepers, and social media (Twitter/X). Participants received a Participant Information Sheet and provided informed electronic consent before participation.

Data collection continued until the research team determined that sufficient conceptual richness had been achieved and that no new concepts relevant to the study objective were being identified [20, 21]. A total of 35 healthcare professionals participated.

### 2.3 Data Collection and Patient and Public Involvement (PPI)

The semi‐structured interview guide was developed collaboratively with Patient and Public Involvement (PPI) contributors to improve clarity, relevance, and alignment with public priorities. PPI contributors were recruited through NHS Research Innovation Groups, the NIHR (National Institute for Health and Care Research) “People in Research” platform and the University of Worcester’s IMPACT: People with Lived Experience groups. PPI contributors included individuals with lived experience of obesity. Feedback informed refinement of the interview schedule, and a pilot interview resulted in minor adjustments before data collection.

The following consent, participants were allocated to individual or group interviews according to participant preference, availability and scheduling. Interviews were audio recorded, anonymized during transcription, and securely stored in accordance with the university’s data management and information security policies. Participants were assigned unique identification numbers, and pseudonyms were used throughout reporting.

### 2.4 Data Analysis

Data were analysed using an inductive reflexive thematic analysis, following the Braun and Clarke reflexive thematic analysis approach [20] and the practical guidance described by Naeem et al. [22]. Transcripts were read repeatedly to foster familiarity before being inductively coded by the first author. Codes were iteratively refined and organized into candidate themes that reflected shared conceptual patterns across the dataset. Themes were subsequently reviewed and refined through reflective discussions within the research team to enhance conceptual coherence and ensure alignment with the study objective. The final themes represented the conceptual foundations underpinning healthcare professionals’ knowledge, attitudes, and beliefs regarding adult obesity and informed subsequent outcome measurement instrument development.

### 2.5 Trustworthiness

Trustworthiness was enhanced through repeated familiarization with the data, iterative coding, and ongoing reflective discussions among the research team, supporting analytical rigor and reflexivity throughout the analytical process. The inclusion of healthcare professionals from multiple disciplines and healthcare settings enhanced the breadth of perspectives relevant to the intended cross‐professional application of the planned outcome measurement instrument. Contributions from patients and the public further enhanced the relevance and clarity of the interview materials prior to data collection.

## 3 Results

### 3.1 Participant Characteristics

Thirty‐five healthcare professionals from multiple disciplines and United Kingdom (UK) care settings participated. Participants varied in professional experience, exposure to obesity care, and role emphasis. Participant characteristics are presented in Table S1.

### 3.2 Overview of Conceptual Themes

Analysis identified four interrelated conceptual themes underpinning healthcare professionals’ knowledge, attitudes, and beliefs regarding adult obesity.

1. Clinical Working Environment: Contextual and organizational determinants of clinical practice
2. Knowledge of Obesity: Conceptual and applied knowledge of obesity
3. Attitudes to Obesity: Affective and interpersonal attitudes towards adults living with obesity.
4. Beliefs about Roles: Beliefs about professional roles, responsibilities and agency.

These themes form the conceptual foundation for the development of a future outcome measurement instrument. Additional illustrative quotations are provided in Table S2.

### 3.3 Theme 1—Clinical Working Environment: Contextual and Organizational Determinants of Clinical Practice

Participants consistently described the clinical working environment as a key influence on how obesity was understood and managed in practice. Organizational systems, resource availability, workplace culture, and role expectations shaped participants’ confidence, communication, and willingness to engage in obesity‐related care. These contextual influences extended beyond individual knowledge or attitudes, affecting how healthcare professionals enacted obesity care within everyday clinical practice.

Structural constraints were frequently identified as barriers to delivering safe and equitable care. Participants described how limited time, staffing, space, equipment, and infrastructure increased the complexity of caring for adult patients living with obesity and constrained clinical decision making.

Managing the airway can be more complex … we might need additional equipment, more staff, or more experienced personnel… everything contributes to the complexity and duration of the procedure.

(P129)

Participants also described how organizational culture influences obesity care. Supportive teams promoted respectful and appropriate care, whereas poor communication and inappropriate workplace behaviors undermined patient dignity and confidence.

She appreciated that the staff were already aware of how to meet her needs, that we weren't shocked by her body composition, and that we were prepared.

(P108)

Finally, uncertainty regarding professional roles, limited training, fragmented care pathways, and restricted access to specialist services reduced participants’ confidence in addressing obesity and contributed to ambiguity regarding responsibility for obesity management.

Overall, the clinical working environment emerged as the principal contextual domain influencing how healthcare professionals’ knowledge, attitudes and beliefs regarding adult obesity were expressed in clinical practice and therefore represents a fundamental domain for future outcome measurement instrument development. Additional illustrative quotations are provided in Table S2.

### 3.4 Theme 2—Knowledge of Obesity: Conceptual and Applied Knowledge of Obesity

Participants described varying levels of knowledge regarding adult obesity, shaped by professional training, clinical experience, and workplace exposure. Although obesity was widely recognized as a complex, multifactorial condition, participants differed in their conceptual understanding, awareness of evidence‐based guidance, and confidence in applying recommended approaches in routine practice. Knowledge was therefore described not simply as factual understanding but as the ability to translate evidence into clinical decision‐making within the realities of everyday healthcare.

Obesity is so complex… there are so many factors at play… it's such an individual thing for each person.

(P113)

While many participants demonstrated awareness of national guidance, including NICE CG189 [7], they frequently reported uncertainty regarding its practical implementation. Clinical practice was often influenced by local organizational policies, available resources, and service constraints, which limited the consistent application of evidence.

we must consider NICE…. However, we are ultimately bound by the trust's guidelines and the resources available.

(P108)

Participants also described variable levels of confidence in managing obesity in clinical practice. Concerns regarding patient safety, limited consultation time, and their own perceived expertise reduced confidence in initiating or sustaining obesity‐related discussions. Several participants acknowledged that these barriers influence both their willingness and ability to provide obesity care. They also recognized that cultural perspectives influenced understandings of body size and health, highlighting that knowledge of obesity is shaped by both clinical evidence and broader social and cultural contexts.

Overall, this theme demonstrates that knowledge extends beyond awareness of obesity or clinical guidelines. Participants consistently described knowledge as context‐dependent, requiring confidence, organizational support, and practical opportunities to apply evidence in routine clinical practice. These findings provide an empirical foundation for the development of knowledge‐related measurement constructs within a future outcome measurement instrument. Additional illustrative quotations are provided in Table S2.

### 3.5 Theme 3—Attitudes Towards Obesity Care: Emotional, Interpersonal, and Cultural Influences

Participants described attitudes towards adults living with obesity as being shaped by emotional, interpersonal, and cultural influences. Although they consistently expressed a desire to provide respectful and compassionate care, many also described uncertainty, anxiety, and frustration when discussing weight. These responses reflected not only personal attitudes but also concerns about damaging therapeutic relationships, using inappropriate language, or failing to meet patients’ individual needs.

You don't want to say the wrong thing….it’s so sensitive.

(P117)

Participants frequently described adapting their language to avoid terms they perceived as stigmatizing. While this was viewed as a way of maintaining empathy and preserving rapport, several participants acknowledged uncertainty about how to discuss obesity openly without causing offense.

I never use the terminology morbid obesity. That just sounds cruel.

(P102)

Attitudes were also influenced by wider cultural beliefs about body size and health. Participants recognized that dominant obesity frameworks did not always reflect the cultural backgrounds or lived experiences of adults, who they argued required greater sensitivity and flexibility in communication.

Overall, this theme suggests that attitudes towards obesity extended beyond individual opinions or bias. They are shaped by interpersonal relationships, communication practices, and broader cultural influences, highlighting the importance of assessing both emotional responses and contextual factors when developing future outcome measurement instruments. Additional illustrative quotations are provided in Table S2.

### 3.6 Theme 4—Beliefs About Roles: Beliefs About Professional Identity, Responsibility and Agency in Obesity Care

Participants expressed diverse beliefs regarding their role in obesity management, the distribution of responsibility across healthcare services, and their ability to influence patient outcomes. These beliefs were shaped by professional identity, organizational expectations, and perceptions of the support available to address obesity within routine clinical practice. Collectively, participants described uncertainty about where responsibility for obesity management should lie and whether they had the authority, resources, or capability to deliver effective care.

Many participants questioned whether obesity management was part of their professional remit, frequently linking this uncertainty to limited training, role expectations, and the absence of obesity within professional curricula. Obesity management was often perceived as secondary to immediate clinical priorities or better suited to other services within the healthcare pathway.

there hasn't been an amendment to our job specifications or our training to factor that in… the curriculum doesn’t cater for that.

(P101)

Participants also expressed differing beliefs regarding responsibility for obesity management. While some viewed obesity care as a shared multidisciplinary responsibility, others considered it primarily the responsibility of general practitioners, specialist services, or the individual patient. These differing perspectives reflect uncertainty regarding accountability across the obesity care pathway.

The first thing they have to really demonstrate is ownership of the problem.

(P123)

Even when participants recognized obesity as an important clinical issue, many described feeling constrained in their ability to intervene effectively. Limited consultation time, restricted access to specialist services, and insufficient organizational resources reduced confidence in addressing issues and contributed to a perception that opportunities for meaningful intervention were often missed. Several participants also described uncertainty regarding how to initiate conversations about weight or support behavioral change without risking conflict or complaints.

Overall, this theme demonstrates that professional beliefs about obesity management extend beyond individual motivation or confidence. Participants’ sense of professional agency was shaped by organizational structures, role clarity, and perceived service capacity, reinforcing the importance of considering contextual influences when developing future outcome measurement instruments. Additional illustrative quotations are provided in Table S2.

## 4 Discussion

This qualitative study provides a novel conceptualization of healthcare professionals’ knowledge, attitudes, and beliefs regarding adult obesity, demonstrating that these constructs are not solely individual attributes but are also shaped by the clinical working environment in which care is delivered. Four interrelated domains were identified: the clinical working environment, knowledge of obesity, attitudes towards obesity, and beliefs about professional roles. Notably, the findings indicate that the clinical working environment serves as a contextual domain that influences how knowledge is interpreted, how attitudes are expressed, and how professional beliefs about responsibility and agency are enacted in practice. This research extends existing knowledge, attitudes, and beliefs literature and stigma‐focused frameworks by situating healthcare professionals’ perspectives within organizational and service contexts, thereby offering a more comprehensive conceptual foundation for the development of outcome measurement instruments to assess adult obesity‐related care.

A key finding is the central role of the clinical working environment in determining how healthcare professionals engage with adult obesity in practice. Structural constraints, such as time pressures, inadequate equipment, limited staffing capacity, and service fragmentation, were consistently identified as factors influencing healthcare professionals' clinical behavior and decision‐making. Furthermore, broader healthcare system structures, including funding arrangements, may also influence access to evidence‐based obesity interventions and specialist services. These wider system‐level factors further emphasize the importance of considering organizational and contextual influences when developing outcome measurement instruments for use across different healthcare systems. Additionally

Additionally, organizational culture played a significant role in shaping whether healthcare professionals proactively addressed or avoided engaging with adults living with obesity, often due to perceived sensitivity, interpersonal risk, or uncertainty. These findings align with implementation science evidence demonstrating that clinicians' behavior is strongly shaped by organizational context [23, 24, 25, 26]. This perspective provides a more comprehensive explanatory framework than existing stigma‐focused reviews, emphasizing the role of system‐level determinants in shaping obesity‐related clinical practices [11, 27].

Healthcare professionals acknowledge that obesity is a multifactorial condition; however, many express a lack of confidence in translating this understanding into effective clinical practice. The NICE guidance (CG189) [7] is often referenced but is frequently regarded as aspirational. Implementation is challenged by competing demands and resource limitations, a situation well‐documented in prior research on barriers to guideline implementation [23, 24, 28]. Knowledge, therefore, should be viewed not only as awareness of guidelines but also as the ability to access, interpret, and apply these recommendations within the constraints of the working environment. This distinction emphasizes the importance of differentiating between theoretical understanding and context‐dependent practical skills when assessing healthcare professionals’ preparedness to manage adult obesity. Moreover, it aligns with previous research indicating that knowledge alone is insufficient to foster changes in clinical behavior without adequate organizational support [26, 29].

Healthcare professionals frequently describe emotional discomfort and anxiety when discussing obesity with adults, fearing that they may offend patients or harm therapeutic relationships. Avoidance terms such as “obese” were often framed as compassionate but may contribute to inconsistencies in care. These findings extend existing evidence demonstrating that weight stigma in healthcare is shaped not only by individual bias but also by organizational and cultural norms [11, 27]. Cultural differences in views on body size and health further influenced communication practices, underscoring that attitudinal responses are socially and contextually embedded.

Beliefs about professional identity and legitimacy impacted whether obesity management was viewed as part of clinicians’ responsibilities. Role ambiguity, fragmented accountability, and inadequate structures within intervention pathways diminished perceived professional agency. These findings echo the broader literature, which emphasizes how organizational clarity and leadership support affect clinician engagement in complex care delivery [25, 28, 30]. Without systemic reinforcement, obesity management may be deprioritized, even when individual clinicians recognize its significance.

These findings provide an empirical foundation for developing a context‐sensitive outcome measurement instrument to assess healthcare professionals’ knowledge, attitudes, and beliefs regarding adult obesity. This emphasis on concept elicitation and content validity is consistent with established measurement theory, which recognizes that robust outcome measurement instruments must be grounded in clearly defined constructs before subsequent psychometric evaluation can be meaningfully undertaken [31, 32, 33]. By focusing on healthcare professionals and the contextual determinants of practice, this study extends existing stigma‐focused research [11, 34]. It highlights the importance of integrating organizational, cultural, and policy influences within measurement frameworks. Outcome measurement instruments which overlook these contexts may misrepresent healthcare professionals preparedness and practices. Future item development should be context‐sensitive, taking into account organizational environments and interpersonal dynamics, to enhance the validity and utility of the instruments across various settings.

The findings also have important implications for the development of a context‐sensitive outcome measurement instrument. Qualitative themes were synthesized to inform a preliminary conceptual framework for future item development. Rather than representing isolated constructs, the findings demonstrated that healthcare professionals’ knowledge, attitudes and beliefs regarding adult obesity were consistently influenced by the organizational and clinical contexts in which care was delivered. Table 1 summarizes how the qualitative themes were translated into the proposed measurement domains to support future item generation.

**TABLE 1:** Mapping of inductively derived qualitative themes to proposed measurement domains.

Qualitative themes	Proposed measurement domain	Core conceptual domains	Measurement focus
Clinical working environment	Clinical working environment	Structural constraints; organizational culture; policy and role clarity	Organizational influences shaping obesity‐related clinical practice
Knowledge of obesity	Knowledge	Conceptual understanding; guideline awareness; practical management confidence	Cognitive understanding and application of obesity‐related knowledge
Attitudes towards obesity care	Attitudes	Emotional responses; language and communication; cultural perceptions	Emotional and interpersonal responses to obesity
Beliefs about roles	Beliefs	Professional role legitimacy; responsibility distribution; behavioral confidence	Professional beliefs influencing engagement in obesity‐related care

Across all interviews, the clinical working environment emerged as the overarching contextual domain influencing healthcare professionals’ knowledge, attitudes, and beliefs. Structural constraints, organizational culture, and role clarity shaped how participants understood obesity, communicated with adults with obesity, and perceived their professional responsibilities. These findings suggest that healthcare professionals' knowledge, attitudes and beliefs are not solely individual characteristics but are context‐dependent constructs embedded within routine clinical practice.

The proposed measurement domains were developed inductively from the qualitative analysis rather than derived from an existing theoretical framework. Table 2 summarizes the proposed measurement domains and their conceptual definitions, while Table S3 provides a more detailed mapping from the qualitative findings to the conceptual domains and associated measurement implications that will inform future item generation.

**TABLE 2:** Conceptual definitions of the proposed measurement domains are derived from qualitative themes.

Proposed measurement domain	Conceptual definition
Clinical working environment	The interplay of structural, cultural, and policy influences that shape obesity‐related practices.
Knowledge	The understanding and application of obesity‐related evidence and guidance.
Attitudes	Emotional and evaluative responses to adults living with obesity.
Beliefs	Professional perceptions of role, responsibility and impact.

This conceptual framework extends previous Knowledge‐Attitudes‐Practice models by recognizing beliefs as a distinct construct and by explicitly incorporating the clinical working environment as a contextual determinant influencing healthcare professionals’ knowledge, attitudes and beliefs [35, 36]. These findings provide an empirical foundation for developing a context‐sensitive outcome measurement instrument for adult obesity care.

### 4.1 Strengths and Limitations

The study encompassed a diverse array of healthcare professionals from various disciplines and care settings across the UK. By utilizing both individual and group interviews, the research highlighted common norms and personal experiences. Patient and public representatives enhanced the study's relevance. However, the recruitment may have biased the sample towards individuals with a particular interest in obesity and may not fully reflect the breadth of professional roles involved in adult obesity care, with some specialties, such as endocrinology, being underrepresented. The use of virtual interviews also posed challenges for participants who were unfamiliar with Microsoft Teams or preferred in‐person interactions.

## 5 Conclusion

This qualitative study underscores that healthcare professionals’ knowledge, attitudes and beliefs towards adult obesity are shaped by the clinical environments in which they operate. Structural constraints, organizational culture, and role clarity significantly influence clinicians’ confidence and communication in adult obesity care. These findings provide a strong empirical foundation for developing a context‐sensitive outcome measurement instrument to support workforce development, service evaluation, and policy implementation within UK healthcare, while providing a conceptual framework to inform future cross‐cultural adaptation and validation across other healthcare systems.

## Conflicts of Interest

The authors declare no conflicts of interest.

## Patient and Public Involvement (PPI) Statement

The interview guide was crafted in collaboration with Patient and Public Involvement (PPI) contributors to ensure clarity, relevance, and alignment with PPI priorities. PPI contributors were recruited through NHS Research Innovation Groups, the NIHR “People in Research” platform, and the University of Worcester’s IMPACT: People with Lived Experience groups. All materials were presented in plain English. Following their review of the interview schedule, the PPI contributors offered feedback on clarity and relevance. A pilot interview was conducted to assess the flow and comprehensibility, resulting in minor revisions before the data collection phase.

## Supporting information


**Table S1:** Participant characteristics.

**Table S2:** Additional illustrative quotations organized by theme and conceptual domain.

**Table S3:** Conceptual definitions informing outcome measurement instrument development.

## Data Availability

To minimize the risk of participant identification, the qualitative data generated during this study are not publicly available but are available from the corresponding author upon request.
