# Supplementary material for: UK Healthcare Professionals’ Knowledge, Attitudes, and Beliefs About Adult Obesity: A Qualitative Study
Source: Obes Sci Pract. 2026 Aug 4;12(4):e70182. doi: 10.1002/osp4.70182 (PMC13435368; doi:10.1002/osp4.70182)
Supplement: Supplementary file 1 [file OSP4-12-e70182-s001.docx]

**SupplementarySupplementary Table 3: Conceptual Definitions informing outcome measurement instrument development.**

| **THEME** | **CONCEPTUAL DOMAIN** | **CONCEPTUAL DEFINITION** | **MEASUREMENT IMPLICATION** |
| --- | --- | --- | --- |
| **Clinical Working Environment** | Structural Constraints | Organisational and physical constraints (e.g., time, space, equipment, staffing) that impact the feasibility and quality of obesity-related care. | Items should assess perceived resource adequacy and environmental barriers affecting obesity related practice. |
|  | Organisational and Cultural Influences | Collective norms, behaviours, and workplace practices that influence the perception and management of obesity within clinical teams. | Items should capture perceived cultural norms and team-level attitudes influencing engagement with obesity care. |
|  | Policy and Role Clarity | Clarity and consistency in policies, job expectations, and care pathways related to obesity management. | Items should assess perceived legitimacy, formal responsibility, and clarity of service pathways. |
| **Knowledge** | Conceptual Understanding of Obesity | Understanding obesity as a multifactorial condition that includes biological, psychological, and social contributors. | Items should distinguish between biomedical knowledge and broader biopsychosocial understanding. |
|  | Guideline Awareness and Application | Awareness and practical application of evidence-based guidelines for obesity management in clinical practice. | Items should assess both awareness and perceived applicability within local contexts. |
|  | Practical Management Confidence | Confidence in applying obesity-related knowledge safely and effectively in day-to-day clinical encounters. | Items should capture applied knowledge rather than mere theoretical awareness. |
| **Attitudes** | Emotional and Cognitive Responses | Affective reactions that influence engagement with obesity-related discussions. | Items should assess emotional comfort and perceived interpersonal risk in discussing obesity. |
|  | Language and Communication | Preferences and behaviours related to terminology, framing, and communication about obesity. | Items should examine communication comfort and concerns related to terminology. |
|  | Cultural Perceptions | Recognition that cultural norms regarding body weight and health shape perceptions, interpretations, and clinical interactions. | Items should assess awareness of cultural influences on obesity-related discussions. |
| **Beliefs** | Professional Role Legitimacy | Beliefs regarding whether obesity management falls within one's professional role. | Items should assess perceived legitimacy and scope of role in obesity care. |
|  | Responsibility Distribution | Beliefs about how responsibility for obesity management should be shared among professionals, services, and patients. | Items should examine perceived accountability and role boundaries. |
|  | Perceived Effectiveness | Beliefs concerning the potential impact of one’s professional role on obesity-related outcomes. | Items should assess perceived efficacy and influence. |
|  | Behavioural Confidence | Confidence in influencing behaviour change and initiating conversations related to obesity. | Items should assess confidence in initiating and sustaining behaviour change discussions, distinct from knowledge content alone. |
