# Supplementary material for: UK Healthcare Professionals’ Knowledge, Attitudes, and Beliefs About Adult Obesity: A Qualitative Study
Source: Obes Sci Pract. 2026 Aug 4;12(4):e70182. doi: 10.1002/osp4.70182 (PMC13435368; doi:10.1002/osp4.70182)
Supplement: Supplementary file 2 [file OSP4-12-e70182-s002.docx]

**Participant Characteristics**

| **Occupation** | **ID** | **Clinical Setting** | **Locality** | **Interview Type** |
| --- | --- | --- | --- | --- |
| Physiotherapist | *101* | Community Care | England, UK | Individual |
| Dietician | *102* | Primary Care | England, UK | Individual |
| Chiropractor | *103* | Primary Care | England, UK | Individual |
| Nurse | *104* | Primary Care | England, UK | Individual |
| Nurse | *106* | Secondary Care | England, UK | Individual |
| Occupational Therapist | *105* | Community Care | England, UK | Individual |
| Physiotherapist | *107* | Community Care | England, UK | Individual |
| Occupational Therapist | *108* | Community Care | England, UK | Individual |
| General Practitioner | *109* | Primary Care | England, UK | Group B |
| General Practitioner | *110* | Primary Care | England, UK | Group B |
| General Practitioner | *111* | Primary Care | England, UK | Group B |
| Nurse | *112* | Primary Care | England, UK | Group B |
| General Practitioner | *113* | Primary Care | England, UK | Group B |
| General Practitioner | *114* | Primary Care | England, UK | Group B |
| Physiotherapist | *115* | Secondary Care | England, UK | Group A |
| Nurse | *116* | Community Care | England, UK | Group A |
| Social Worker | *117* | Community Care | England, UK | Group A |
| Social Worker | *118* | Community Care | England, UK | Group A |
| Nurse | *119* | Community Care | England, UK | Group A |
| Social Worker | *120* | Community Care | England, UK | Group A |
| Occupational Therapist | *122* | Community Care | England, UK | Individual |
| Consultant Orthopaedic Surgeon | *123* | Secondary Care | England, UK | Individual |
| Consultant Orthopaedic Surgeon | *124* | Secondary Care | England, UK | Individual |
| Physiotherapist | *126* | Community Care | England, UK | Individual |
| Dietician | *127* | Community Care | England, UK | Individual |
| Consultant Anaesthetist | *128* | Secondary Care | Scotland, UK | Individual |
| Consultant Anaesthetist | *129* | Secondary Care | Scotland, UK | Individual |
| Physiotherapist | *130* | Secondary Care | England, UK | Individual |
| Physiotherapist | *131* | Secondary Care | England, UK | Individual |
| Consultant Anaesthetist | *132* | Secondary Care | Scotland, UK | Individual |
| General Practitioner | *133* | Primary Care | Scotland, UK | Individual |
| Physiotherapist | *134* | Secondary Care | England, UK | Group C |
| Radiotherapist | *135* | Secondary Care | Wales, UK | Group C |
| Radiotherapist | *136* | Secondary Care | Wales, UK | Group C |
| Physiotherapist | *137* | Primary Care | England, UK | Group C |
