# Supplementary material for: UK Healthcare Professionals’ Knowledge, Attitudes, and Beliefs About Adult Obesity: A Qualitative Study
Source: Obes Sci Pract. 2026 Aug 4;12(4):e70182. doi: 10.1002/osp4.70182 (PMC13435368; doi:10.1002/osp4.70182)
Supplement: Supplementary file 3 [file OSP4-12-e70182-s003.docx]

**Supplementary Table 2. Additional illustrative quotations organised by theme and conceptual domain.**

**Theme 1: CLINICAL WORKING ENVIRONMENT**

| **Conceptual Domain** | **Quotation** | **Participant ID** |
| --- | --- | --- |
| Structural constraints | *"I believe that health and social care systems can be detrimental to individuals who are morbidly obese. There are often significant challenges related to equipment provision and obtaining funding for the appropriate resources. The issues surrounding moving and handling can also be more complex for them."* | *122* |
| Structural constraints | *"Managing the airway can be more complex as well, which means we might need additional equipment, more staff, or more experienced personnel. It’s important to consider all of the people in the operating room, the equipment available, and the time dedicated to each individual task, along with the overall operating room setup. Everything contributes to the complexity and duration of the procedure."* | *129* |
| Structural constraints | *"You know, because they end up taking two bed spaces because of all their things, like the equipment for the gantry hoist and specialist slings. And, you know, large walking aids and things like that. They take up two bed spaces, but then they become a spectacle."* | *107* |
| Structural constraints | *"It's really about how I can safely manage a patient and provide them with the best care possible, especially considering the challenges that obesity presents. Tasks that we often take for granted, such as inserting a cannula, can be quite difficult and may significantly increase the time required for patient care—time that you typically wouldn’t account for at all."* | *129* |
| Structural constraints | *"The fit of blood pressure cuffs. Standard cuffs are designed for arms that are conical in shape, but many larger patients have arms that are more cylindrical. As a result, blood pressure cuffs may not fit properly, leading to inaccurate readings."* | *132* |
| Organisational and cultural influences | *"Patients come in with specific concerns, such as seeing a doctor for a knee injury or discussing mental health issues, and they have a clear idea of the doctor's role. When doctors or other clinicians start discussing broader health topics, patients can feel confused or frustrated, especially if they came in expecting to address a particular problem, like a cut on their arm or an acute mental health episode."* | *120* |
| Organisational and cultural influences | *"The influence of social media has caused patient expectations to increase. People now expect to be seen and addressed quickly, and this can create pressure to resolve issues promptly rather than taking a more holistic approach to health."* | *120* |
| Organisational and cultural influences | *"I think there’s a sensitivity involved here. Patients are already coming to me because they’re dealing with cancer, and my main focus is on caring for them. It feels a bit out of place to suggest something like, 'By the way, while you’re receiving cancer treatment, have you ever thought about losing some weight?' They are likely already aware of their situation."* | *135* |
| Organisational and cultural influences | *"The patient will come in defensive because they're ready because they've had it forever. We've got you come in for one thing, and we go about, even if it's not related to that particular thing. We'll still do it because we've been, we've got this thing of making every contact count, so we must address that well, actually."* | *104* |
| Organisational and cultural influences | *"it almost like a zoo attraction in a way which was heartbreaking because it, at the end of the day, this is a person."* | *105* |
| Organisational and cultural influences | *"These cases presented significant challenges regarding our moving and handling techniques and our ability to help them achieve their rehabilitation goals. However, I genuinely feel that we made substantial progress. We had to learn new handling skills, collaborate with equipment representatives, and engage in complex discharge planning. This included liaising with community teams and conducting access and pre-discharge visits."* | *107* |
| Organisational and cultural influences | *"'How heavy is he now? Is he still 58 stone?' I thought, 'This is confidential information that should not be circulating among staff members. Such details should not be known by someone from the Estates team who is walking around the ward. It’s unacceptable.'"* | *107* |
| Organisational and cultural influences | *"For personal care, we understand that two people may not be enough; often, a third person is necessary to assist with tasks like rolling the patient or applying different creams. Our experience at Malvern with numerous bariatric patients has equipped us well for the challenges and requirements involved."* | *108* |
| Organisational and cultural influences | *"Once she got to the hospital, they lacked the facilities to help her out of bed. By the time she finally arrived at our facility, she was just grateful to have the opportunity to engage in some therapy and to sit up. She appreciated that the staff were already aware of how to meet her needs, that we weren't shocked by her body composition, and that we were prepared to care for her."* | *108* |
| Policy and role clarity | *"we don't because there hasn't been an amendment to our job specifications or our training to kind of factor that in. And I certainly don't think from previous working at the university that the undergraduate postgraduate curriculum caters for that"* | *101* |
| Policy and role clarity | *"At *****, the guidelines suggest that if the multidisciplinary team (MDT) determines that a patient needs to lose weight first, they will route the patient to the obesity clinic afterwards for proper care."* | *106* |
| Policy and role clarity | *"New guidelines state that if a patient is obese, they cannot undergo the procedure due to the high risks involved, and their atrial fibrillation is likely to recur."* | *106* |
| Policy and role clarity | *"...the thing I'm finding most frustrating at the moment is the lack of perhaps services that are tailored towards helping obese patients lose weight. We're having issues with everybody wanting injections, for example, like Ozempic or Monjaro, and I think the availability and the criteria for that need looking at."* | *114* |

**Theme 2: KNOWLEDGE**

| **Conceptual Domain** | **Quotation** | **Participant ID** |
| --- | --- | --- |
| Practical management confidence | *"I work with a lot of patients who are overweight and obese yeah but I would say I lack confidence about having these conversations because of my own struggles but again my current role I just feel I don't have time to bring these topics up."* | *116* |
| Practical management confidence | *"I always get them to keep a food and activity diary... Often, it's because they've got no concept of what they're eating and the amount of calories they're taking in... Once you get a few days, you'd be like, well, you're eating 4 to 5000 calories a day—all the wrong things—and then they're telling me they're doing all this exercise. Reality is very different."* | *116* |
| Practical management confidence | *"I am confident in terms of managing them, because I know it's possible. And I've seen people lose weight, so I've seen the positive outcomes. I know the research, so I'm confident in my skill set and know that I'm advising the right things."* | *102* |
| Practical management confidence | *"I've upset people by bringing up weight and I have made people... become angry because I've gone about the wrong way... I don't tend to get that as much now because I'm better at it."* | *104* |
| Practical management confidence | *"I have pursued specific training opportunities related to the management of morbidly obese patients... This training has enhanced my confidence in working within my speciality."* | *122* |
| Practical management confidence | *"I think it would be a disservice not to inform someone that the most effective way to alleviate their pain might be something they can actively control—like managing their weight."* | *123* |
| Conceptual understanding of obesity | *"I think it's what I'm comfortable with, because I know, like with BMI as a black person, that may be different from how other ethnicities measure obesity, because in some cultures it's okay to be a certain weight, while in other cultures, we look at weight being attractive, and others it's not attractive."* | *119* |
| Conceptual understanding of obesity | *"I do know a lot of things from lived experience, where I know about BMI, you know, checking all that, and the things that I do have to do. And sometimes it's really more about sharing some of the approaches that I've used myself... especially around exercise, you know, there is a lot of research around that."* | *115* |
| Conceptual understanding of obesity | *"If we're saying somebody is obese, we're talking about the body fat... Let's look at like a big rugby player. Yeah, they don't have fat, they're lean, they've got a heavy bone. Are you going to classify them as obese and put them on the four pillars of obesity?"* | *119* |
| Conceptual understanding of obesity | *"That's where local frameworks may not always work. Already, we see that it's very Eurocentric... it's not one size fits all."* | *115* |
| Conceptual understanding of obesity | *"Obesity is so complex... there are so many factors at play... it's such an individual thing for each person."* | *113* |
| Conceptual understanding of obesity | *"Until recently... obesity really hasn't been seen as a disease... medical research now is actually showing that there are physiological differences in people's brains, and it is a disease."*  *"I believe there needs to be a significant increase in awareness regarding obesity, as it clearly affects our roles and workloads..."* | *113* |
| Conceptual understanding of obesity | *"The rates of trauma and abuse among severely obese individuals are significantly higher than in the general population... This response can be similar to addiction or drug abuse."* | *132* |
| Guideline awareness and application | *"That's where local frameworks may not always work. Where they cannot be applied everywhere. Already, we see that it's very Eurocentric, you know, it's a Western framework that takes the body weight of people in a certain context. But also, you know, that's a very, very good example of how, you know, you can't, it's not one size fits all."* | *115* |
| Guideline awareness and application | *"I probably couldn't recite that guideline, but I'm aware that Nice is the clinical commissioning group for the NHS and the guidelines for them. I'm aware of the guideline and that it's a multifaceted approach to managing obesity."* | *101* |
| Guideline awareness and application | *"The NICE guidelines and fair access to care, we must consider them in our practice. However, we are ultimately bound by the trust's guidelines and the resources available to us."* | *108* |
| Guideline awareness and application | *"I frequently refer to is the Society for Bariatric Anesthesia, which has published several useful guidelines covering practical aspects as well as specific anesthetic equipment and medications."* | *129* |

**Theme 3: ATTITUDES**

| **Conceptual Domain** | **Quotation** | **Participant ID** |
| --- | --- | --- |
| Emotional and cognitive responses | *"Talking about weight is usually or often associated with negative experiences, especially in my case. I believe it has become less of a focal point in my management of patients as a result. Personally, I think discussing this topic can induce anxiety because it's so sensitive. You don't want to say the wrong thing, but you're really trying to say the right thing. However, depending on the individual and their circumstances, it can be quite challenging."* | *117* |
| Emotional and cognitive responses | *"Some individuals feel that their current health conditions are greatly impacted by their obesity. They may have been told this directly or implied in the past, but they don’t believe they can make a change, which puts them in a challenging cycle. It’s important to engage with these individuals in a supportive way. Sometimes, being upfront about the issue is necessary. We can talk about obesity not as the central issue but rather as a factor affecting their overall quality of life, particularly in relation to specific concerns, such as back pain. So, let's focus on that and look for solutions together."* | *103* |
| Emotional and cognitive responses | *"People are just so upset and distressed with living with obesity, they're really, really struggling and feel guilty on top of having to live with the extra weight and what that does to them. And yeah, the guilt and the shame and the poor self-esteem and all those things really, really emotional, tearful, often tearful."* | *104* |
| Emotional and cognitive responses | *"To me, a patient's a patient, I don't care where they're from, what size they are, what their beliefs are, or what they want to do. I'm there to treat them, and I treat all my patients the same. I just don't understand why other people don't necessarily do that and why a judgment is needed. A patient is there because they're not well. They need help to get better. See that from an acute sickness? Something more long-term? It doesn't matter. They're there for a reason, and they're there for us to help them. And we've trained to help them. So I don't see why we need to judge them for anything. And it just makes me really sad when they are judged by others."* | *105* |
| Emotional and cognitive responses | *"I feel frustrated for them because I believe self-control is essential. However, this issue often ties closely to psychology, as it’s clear they may be dealing with some sort of mental health problem. Until that underlying issue is addressed, it seems like an addiction to me; they are eating for a specific reason."* | *131* |
| Emotional and cognitive responses | *"Many people have strong emotions attached to weight, and I often wonder how much my interventions can really help. It doesn't seem like my discussions make a significant difference, and perhaps that's just my perception."* | *133* |
| Perceived barriers & cultural factors | *"That's where local frameworks may not always work. Where they cannot be applied everywhere. Already, we see that it's very Eurocentric, you know, it's a Western framework that takes the body weight of people in a certain context... you can't, it's not one size fits all."* | *115* |
| Perceived barriers & cultural factors | *"When I was still back home in Africa, I'm quite slim, and I used to be classified as skinny, but when I came here and somebody said to me, 'Oh, you've got a good body structure,' I was surprised... Growing up, my cousins and sisters were curvy and seen as the normal standard, whereas here it was the opposite."* | *118* |
| Perceived barriers & cultural factors | *"Their parents were both morbidly obese as well, and you could see triggers in their life. As bad things happened, their weight increased significantly... an unhealthy relationship with food from childhood, maybe a lack of education, and then comfort eating as life events occurred."* | *105* |
| Perceived barriers & cultural factors | *"While I want to provide the best care possible to each patient, I must also keep an eye on the schedule. The immediate pre-operative period is not the appropriate time for these discussions; there needs to be a more suitable time and place."* | *129* |
| Perceived barriers & cultural factors | *"Many individuals have entrenched patterns of behavior that they struggle to break on their own. Unfortunately, we lack access to counseling services, such as cognitive behavioral therapy, which many could benefit from."* | *108* |
| Language and patient descriptions | *"When we use the clinical term obesity, patients understand it in a socially stigmatised way. So… make it more clinical, don’t tell the patient ‘you’re obese.’"* | *102* |
| Language and patient descriptions | *"I never use the terminology morbid obesity. That just sounds cruel."* | *102* |
| Language and patient descriptions | *"It's not appropriate for me to tell them, 'You need to lose weight.' Instead, I can suggest that it might be easier for them if they were able to lose some weight, or we could consider modifying some equipment to make them more comfortable."* | *122* |
| Language and patient descriptions | *"It takes a lot longer to have a conversation… you have to choose your language carefully."* | *128* |

**Theme 4: BELIEFS**

| **Conceptual Domain** | **Quotation** | **Participant ID** |
| --- | --- | --- |
| Professional Identity & legitimacy | *"We don't [address weight] because there hasn't been an amendment to our job specifications or our training to factor that in… the undergraduate and postgraduate curriculum doesn’t cater for that."* | *101* |
| Professional identity & legitimacy | *"I don't think many patients expect me or my therapy team to be engaged in their issues… our focus is on functional recovery and discharge. Obesity management seems more suited to community-based follow-ups with GPs."* | *130* |
| Professional identity & legitimacy | *"I'm uncertain about what people with a high BMI truly think when they come to see me… I'm not sure they are thinking about their weight at that moment or what I might do to help them with it."* | *129* |
| Professional identity & legitimacy | *"While health promotion is not a primary aspect of my clinical role, there is a growing emphasis in anaesthesiology… but it remains a secondary focus in my practice."* | *132* |
| Professional identity & legitimacy | *"I think we need to take responsibility because I'm unsure who else should. It's a broad issue, and it raises the question: who should address it?"* | *131* |
| Perceived effectiveness of role | *"We have an opportunity to help intervene when someone has a health concern… However, the reality is that we often don't have the time and resources in our jobs to do anything beyond the immediate tasks at hand."* | *129* |
| Perceived effectiveness of role | *"Individuals with obesity may be less responsive to adopting healthy behaviours… personality traits may have contributed to their obesity, making it harder to engage with structured health programs or lifestyle changes."* | *123* |
| Perceived effectiveness of role | *"Once patients are referred to us, we have to confront them directly if they are obese… we cannot offer them any treatment at that point. This means we might have to deal with frustrated individuals who have waited for months or even years for help."* | *124* |
| Perceived effectiveness of role | *"It used to feel like patients wanted a magic wand, but I didn’t have one. Right now, injectables are being treated like that magic wand… I wonder what the future holds."* | *133* |
| Perceived effectiveness of role | *"Although it may not always seem like the most relevant issue at the moment, I believe we should address it… However, often we fail to do so."* | *130* |
| Responsibility distribution | *"I think General Practitioners (GPs) should refrain from referring patients whose BMI is above 40… they essentially pass the issue along."* | *124* |
| Responsibility distribution | *"The first thing they have to really demonstrate is ownership of the problem."* | *123* |
| Responsibility distribution | *"Somebody needs to think about the societal impact of our current diets and how to change that first, perhaps."* | *114* |
| Responsibility distribution | *"Ultimately, it is up to the GP to assess the patient and determine if they meet the criteria for referral to the bariatric service… there is a waiting list of two to three years."* | *124* |
| Confidence in influencing behaviour | *"He was over 120 kilogrammes and he gradually lost weight… I was almost lecturing when I first saw him… that worked with that patient… but for another person, that would lead to a massive complaint."* | *111* |
| Confidence in influencing behaviour | *"You've got this excess weight going through this joint… it will improve your mental health… Sometimes, it works."* | *111* |
| Confidence in influencing behaviour | *"Yeah, it is part of my role to address it… I address it in every consultation unless there’s some very urgent, pressing issue."* | *102* |
| Confidence in influencing behaviour | *"If we observe that patients are motivated… we might consider referring them… with the expectation they will have a lower BMI by the time they see us."* | *124* |
